# Pharmacokinetics and Tissue Distribution of Nasal Spray of a Novel Muscarinic Receptor Blocker, 101BHG-D01, in Dogs and Rats

**DOI:** 10.2174/1389200224666221201123254

**Published:** 2022-12-31

**Authors:** Hao Wei, Lei Wu, Yongliang Jia, Jian Shen, Yanyou Li, Peng Sun, Qiangmin Xie, Xiaoping Chen, Yicheng Xie, Yingshuo Wang, Ziming Zhao

**Affiliations:** 1 Department of Pharmacy, Xuzhou Medical University, Xuzhou, 221004, China;; 2 Department of Pulmonology, The Children’s Hospital, National Clinical Research Center For Child Health, Zhejiang University School of Medicine, Hangzhou, 310052, China;; 3 Key Laboratory of Respiratory Drugs Research, Zhejiang University School of Medicine, Hangzhou, 310058, China;; 4 Beijing Showby Pharmaceutical Co., LTD, Beijing, China;; 5 Innovation Research Institute of Chinese Medicine, Shandong University of Traditional Chinese Medicine, Jinan, 250355, China

**Keywords:** M3 receptor blocker, nasal spray, pharmacokinetics, tissue distribution, cholinergic receptors, Parkinson's disease

## Abstract

**Background:**

101BHG-D01 is a novel selective anti-muscarinic (M) 3 receptor-blocking drug. 101BHG-D01 nasal spray is intended to be used to relieve sneezing and runny nose symptoms caused by allergic rhinitis.

**Methods:**

In this study, we examined the plasma pharmacokinetics, tissue distribution, and major excretion mode of 101BHG-D01 in Beagle dogs and rats following nasal spray and intranasal administration, respectively, using HPLC-MS/MS.

**Results/Discussion:**

We found that the pharmacokinetics of 101BHG-D01 was linear in dogs. 101BHG-D01 entered the bloodstream rapidly following nasal spray. Its plasma half-life was approximately 6 h and resided at least 24 h in the body. Moreover, 101BHG-D01 retained a significant amount in the nasal cavity. Finally, we found that 101BHG-D01 was eliminated mainly in the form of stools in rats.

**Conclusion:**

In conclusion, we provided pertinent reference information regarding the design and optimization of drug delivery regimens for clinical trials.

## 1 INTRODUCTION

Muscarinic (M) receptor blockers are a class of drugs that bind to cholinergic receptors without eliciting cholinergic effects, thereby inhibiting the binding of cholinergic neurotransmitters or cholinergic pharmaceuticals to receptors and inducing anticholinergic effects [1]. They are frequently prescribed to treat a variety of conditions, including Parkinson's disease, depression, cardiovascular disease, urinary incontinence, asthma, and allergic diseases [2-4]. This is due to the fact that they are safe, effective, and reasonably priced. There are a few subtypes of M receptors [5-8]. M1 receptors are predominantly found in sympathetic postganglionic nerve and gastric parietal cells, and their activation results in excitation and gastric acid secretion [9]; M2 receptors are predominantly found in the myocardium, and their activation results in decreased cardiac contractility and heart rate [10], and M3 receptors are predominantly found in smooth muscle and blood vessels [6, 11]. The pharmacological subtypes of the M4 and M5 receptors have not yet been determined [4]. They are classified as selective M receptor blockers or non-selective M receptor blockers based on their selectivity for M receptors. Due to their precise targeting and fewer adverse effects, selective M-receptor blockers have a greater potential for application [12].

Pharmacokinetic research is critical for ensuring the safety and efficacy of novel drugs. It aims to establish useful pharmacokinetic parameters for the absorption, distribution, metabolism, and excretion of drugs in the body. It is critical for preclinical pharmacological research as well as drug development. For many years, M-receptor blockers have been used to treat chronic obstructive pulmonary disease (COPD) and asthma [2, 4]. Numerous studies on the pharmacokinetics of M receptor blockers *in vivo* have been conducted using a variety of administration methods, including intratracheal instillation [12], oral administration [12], and intravenous injection [13]; however, not many studies have been conducted for the pharmacokinetics of nasal spray administration.

Nasal spray administration is highly dependent on the quick absorption of drugs through the nasal mucosa in order to achieve the goal of rapid treatment [14]. Nasal spray administration is also the primary route of administration for allergic rhinitis treatment [15, 16]. It not only directly targets the diseased organ but also successfully avoids the first-pass effect associated with systematic administration, significantly increasing the drug's bioavailability. 101BHG-D01 is a selective M receptor antagonist with the greatest efficacy against the M3 receptor, which is rapidly dissociated from M2 receptors but slowly from M1 and M3 receptors. It may contribute to the amelioration of cardiotoxicity associated with non-selective M2 receptor antagonists [4]. When administered *via* nasal spray, the primary objective of 101BHG-D01’s design is to treat symptoms, such as sneezing and runny nose caused by allergic rhinitis and nasal inflammation following a cold. In another unpublished study, we demonstrated that 101BHG-D01 effectively alleviated the symptoms of allergic rhinitis.

The purpose of this study was to characterize the pharmacokinetics and tissue distribution of 101BHG-D01 following nasal administration in dogs and rats, as well as to determine the predominant route of 101BHG-D01 excretion in rats. Thus, it provides essential reference data for the development and improvement of clinical trials.

## 2 MATERIALS AND METHODS

### 2.1 Animals

All rodents were purchased from Zhejiang Weitong Lihua Laboratory Animal Technology Co., Ltd. (Certificate No.: SCXK (Zhe) 201-0001, Zhejiang Province, China). Beagle dogs were purchased from Changzhou Belle Laboratory Animal Breeding Co., Ltd. (Certificate No.: SCXK (Su) 2018-0007, Jiangsu Province, China). The weight of SD rats and Beagle dogs was controlled at around 250 g and 9 kg, respectively. All animals were housed for 7 days (Beagle dogs) or for 3 days (Rats) to allow them to acclimate before the experiment. All animals were bred under the conditions of 23 ± 3°C with 55 ± 15% humidity and a 12 h light/12 h dark cycle; SD rats were fed in the barrier system animal room of the Key Laboratory of Drug Safety Evaluation and Research of Zhejiang Province at SPF level; Beagle dogs were fed in the animal room of the same institute. Rats were kept for more than 12 h before dissection, and dogs were kept without allowing to water for more than 8 h before drug administration. All the animal care and handling procedures were approved by the Institutional Animal Care and Use Committee of the Key Laboratory of Drug Safety Evaluation and Research of Zhejiang Province.

### 2.2 Compound and Administration

**Fig. (1):**
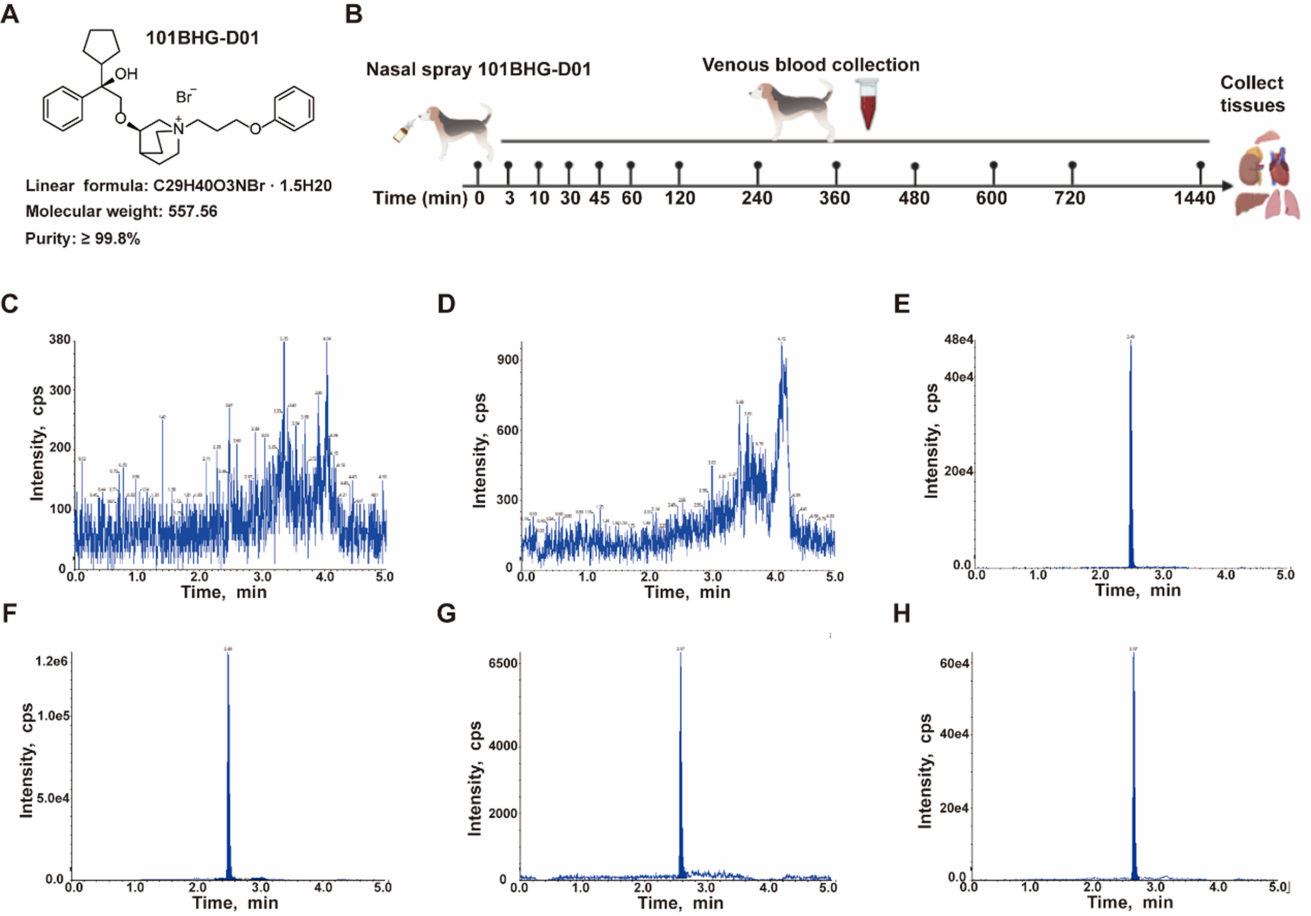
Flow chart of this experiment. **A**) The structural characteristics of the investigated drug - 101BHG-D01. **B**) The flow chart of the experiment. **C** and **D**) A chromatogram of a blank sample. **E**) A chromatogram of blank sample spiked with 101BHG-D01 (200 pg/mL). **F**) A chromatogram of blank plasma sample spiked with IS (200 pg/mL). **G** and **H**) Chromatograms of testing sample. IS: Internal Standard.

101BHG-D01 nasal spray is a colorless, odorless, clear liquid manufactured by Beijing Jiashi Lianbo Pharmaceutical Technology Co., Ltd (Y-AP-19011-1 for rats, Y-AP-19011-4 and Y-AP-19011-5 for Beagle dogs, Beijing Province, China), which is sprayed out in the form of a mist by pressing the valve. It has the same viscosity as water, conforms to United States Pharmacopoeia (USP) dosage accuracy standards, and is stable for up to 24 months at room temperature. Its chemical name is (R)-3-((R)-2-cyclopentyl-2-hydroxy-2-phenylethoxy)-1-(3-phenoxypropyl)-quinuclidin-1-ium bromide sesquihydrate (Figs. **1A** and **B**).

Due to the fact that 101BHG-D01 nasal spray is a fixed-dose nasal spray, the diameter of the nozzle is significantly greater than the diameter of the nostrils of rats; it must be supplied through nasal drop after shaking the liquid (36 µg/kg). The dosage of nasal spray for Beagle dogs is derived and concerted straight from the dog’s weight (Table **1**).

### 2.3 Sampling

The blood was obtained from the subcutaneous vein of the forelimb of the Beagle dogs after shaving and disinfecting the collection site, and 1-1.5 mL of blood was extracted from each animal and placed in a heparin sodium anticoagulation tube. Centrifugation (5,000 rpm, 15 min) was used to isolate plasma, which was then kept at -80°C until analysis.

Tissue samples were taken following anaesthesia and bloodletting. Each animal had a single sample obtained from the same place. A suitable amount of 0.9% sodium chloride solution (1:4, g:mL) was added based on the weight of the sample. After homogenization, it was held at -80°C until testing.

Before and after dosage, stool and urine samples were obtained. After drying and weighing the stool, an adequate volume of methanol and water (1:1) was added to determine the final volume of the additional solution in relation to the mass of the stool (20:1, mL:g), as well as the homogenate after soaking. After collecting urine, the total amount was noted, 1% Tween 80 was added to water (20:80), and then they were mixed thoroughly and stored at -80°C until testing.

### 2.4 HPLC-MS/MS Assay

101BHG-D01 concentration was determined in plasma, tissue, stool, and urine samples utilizing a sensitive and selective liquid chromatography coupled with triple electrospray mass spectrometry (HPLC-MS/MS, AB5500 for plasma; QTRAP 6500+ for tissues, SCIEX AB, USA). HPLC-MS/MS procedures were validated in accordance with current international standards, typically having a calibration range of 10-2,560 or 40-10,000 pg/mL.

### 2.5 Method Validation

#### 2.5.1 Selectivity

The method was selected by analyzing blank biological samples (plasma, tissue homogenates, and excreta), blank biological samples spiked with analytes, internal standard (IS), and biological samples collected following dosing. These samples were used to determine whether endogenous matrix components interfere with experiments and to assess the method's selectivity (Figs. **1C**-**H**).

#### 2.5.2 Linearity and Lower Limit of Quantitation

Using the standard samples, calibration curves were created. The ratio of the analyte and IS peak areas to the ratio of quality control (QC) samples with varying concentrations was plotted using a weighting factor of 1/x^2^; a linear regression equation utilizing the least squares approach was generated. The lower limit of quantification (LLOQ) was determined as the calibration curve's lowest concentration with a signal-to-noise ratio (SNR) was 5. Table **2** summarizes the results.

#### 2.5.3 Accuracy and Precision

Precision was determined in terms of QC sample relative standard deviation (RSD) and relative error (RE), and accuracy was assessed by comparing measured concentrations to actual concentrations.

Four samples of various concentrations of 101BHG-D01 were prepared and operated according to each sample treatment item. Six samples of each concentration and the peak area ratio were substituted into the standard curve of the day to calculate the measured concentration.

If the RSD and RE values were within ± 20%, the method’s precision and accuracy would meet the requirement for measuring biological samples. The experimental results are shown in Table **3**.

#### 2.5.4 Extraction Recovery and Matrix Effect

We investigated recoveries using QC samples with low, medium, and high degrees of contamination. Recovery was calculated using the ratio of spiked analytes’ peak areas in the extracted samples to blank samples of the same concentration.

The matrix effects were determined by comparing the peak regions of an extracted blank sample to those of a pure standard solution containing the analyte in the same concentration. The results are shown in Table **4**.

#### 2.5.5 Stability

The stability of 101BHG-D01 was investigated under a variety of storage and processing settings, including at room temperature (17 h for plasma, 46 h for tissue, 43 h for excrement), 3 freeze-thaw cycles, and long-term freezing at -70°C, and stored in an autosampler for 24 h. The results are shown in Table **5**.

#### 2.5.6 Pharmacokinetic Analysis

Pharmacokinetic parameters were calculated using DAS software (3.0, Shanghai University of Traditional Chinese Medicine, CHN) based on the blood concentration. The pharmacokinetic parameters of the low, medium, and high dose groups are shown in Table **6**.

### 2.6 Statistical Analysis

Statistical analysis was performed using GraphPad Prism software (GraphPad Software, USA). All data were expressed as mean ± SD. When the data were multiple sets of samples, significance was tested by one-way ANOVA followed by Dunnett's multiple comparisons test. When the data were paired samples, significance was tested by an unpaired t-test followed by Welch's correction test for comparison between groups. *p* < 0.05 was considered statistically significant.

## 3 RESULTS

### 3.1 Pharmacokinetics of 101BHG-D01 in Dogs After Nasal Spray

**Fig. (2):**
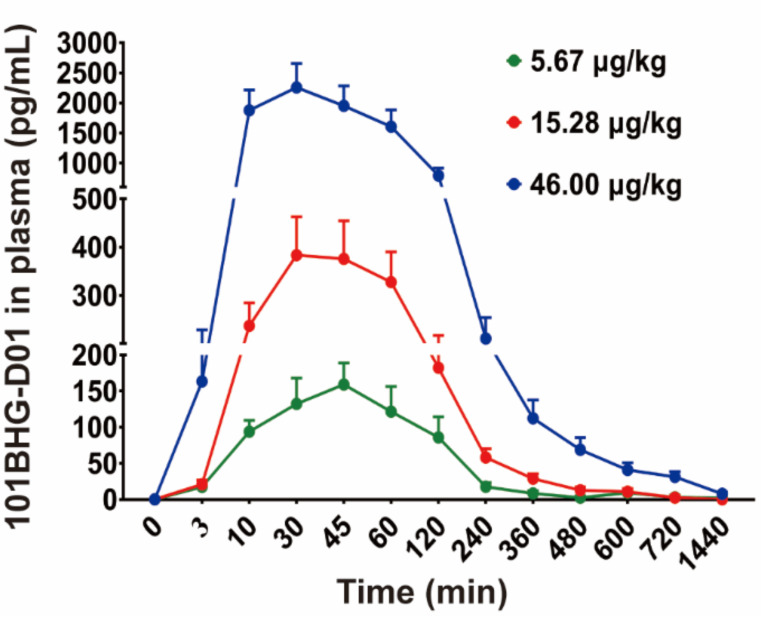
101BHG-D01 rapidly entered the bloodstream following nasal spray in dogs. After a nasal spray of 101BHG-D01, the plasma concentration-time curve was determined in dogs. The results are shown as mean ± SD, n = 6/group.

**Fig. (3):**
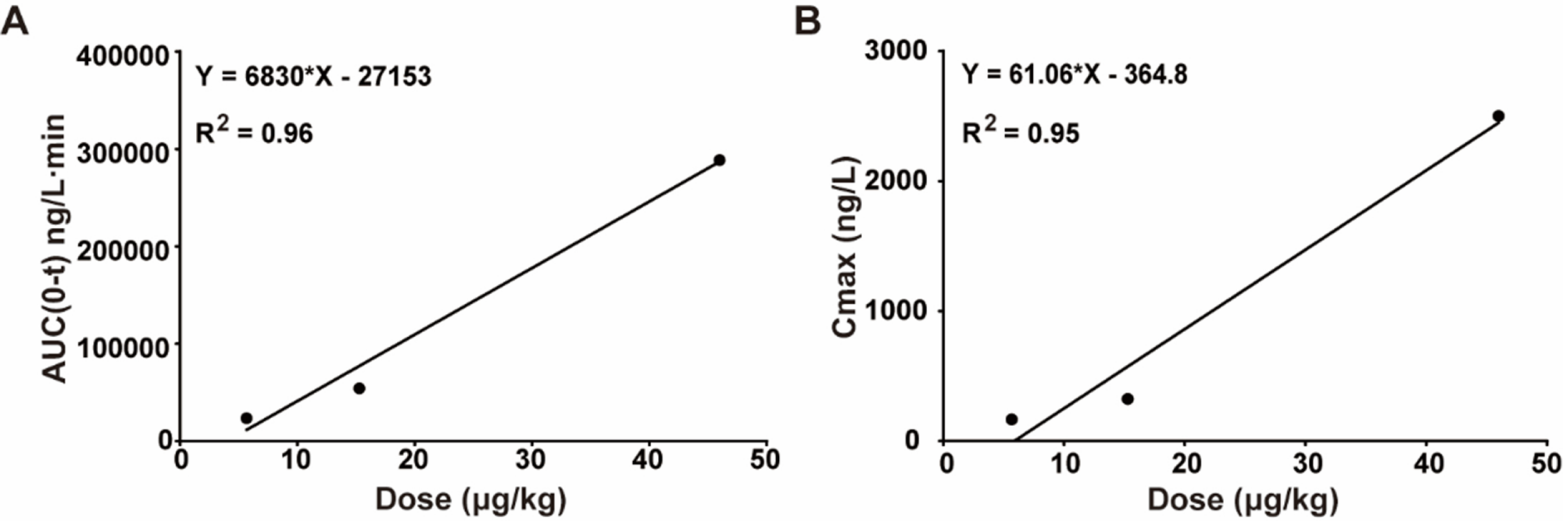
The pharmacokinetics of 101BHG-D01 nasal spray in dogs is a linear kinetic process. **A**) There is a linear correlation between AUC (0-t) and dosage. **B**) There is a linear correlation between Cmax and dosage. The results are shown as mean ± SD, n = 6/group. An equation was made by linear regression analysis. Correlation analysis was performed by computing Pearson’s correlation coefficient.

The pharmacokinetics of 101BHG-D01 was determined in dogs using a variety of dosages delivered through nasal spray (Fig. **1** and Table **1**). The 101BHG-D01 concentration-time curve was created using the plasma drug concentration at various time intervals (Fig. **2**). The pharmacokinetic parameters associated with the curve are reported in Table **6**. The drug reached the bloodstream at the initial point of observation as expected (3 min). The concentration peaked between 10 and 60 min after treatment (5.67 µg/kg: 42 ± 13min, 166.75 ± 69.66 ng/L; 15.28 µg/kg: 33 ± 6 min, 389.98 ± 192.68 ng/L; 46.00 µg/kg: 29 ± 11 min, 2,371.91 ± 964.26 ng/L; Fig. **2**, Table **6**). 101BHG-D01 retained around 150 min in dog plasma (MRT, 5.67 µg/kg: 107.97 ± 62.11 min; 15.28 µg/kg: 124.49 ± 15.84 min; 46.00 µg/kg: 147.04 ± 27.47 min; Table **6**), and clearance was roughly 0.2 L/min/kg (CLz/F, 5.67 µg/kg: 0.17 ± 0.06 L/min/kg; 15.28 µg/kg: 0.34 ± 0.25 L/min/kg; 46.00 µg/kg: 0.22 ± 0.19 L/min/kg; Table **6**). Finally, we determined that the AUC (0-t) and Cmax values of 101BHG-D01 in dog plasma were linearly related to the dose (AUC 0-t: R^2^ = 0.96, Cmax: R^2^ = 0.95 Fig. **3A** and **B**) through correlation analysis, showing that 101BHG-D01 had linear kinetics in dogs.

### 3.2 Tissue Distribution of 101BHG-D01 in Dogs 24 h After Nasal Spray

**Fig. (4):**
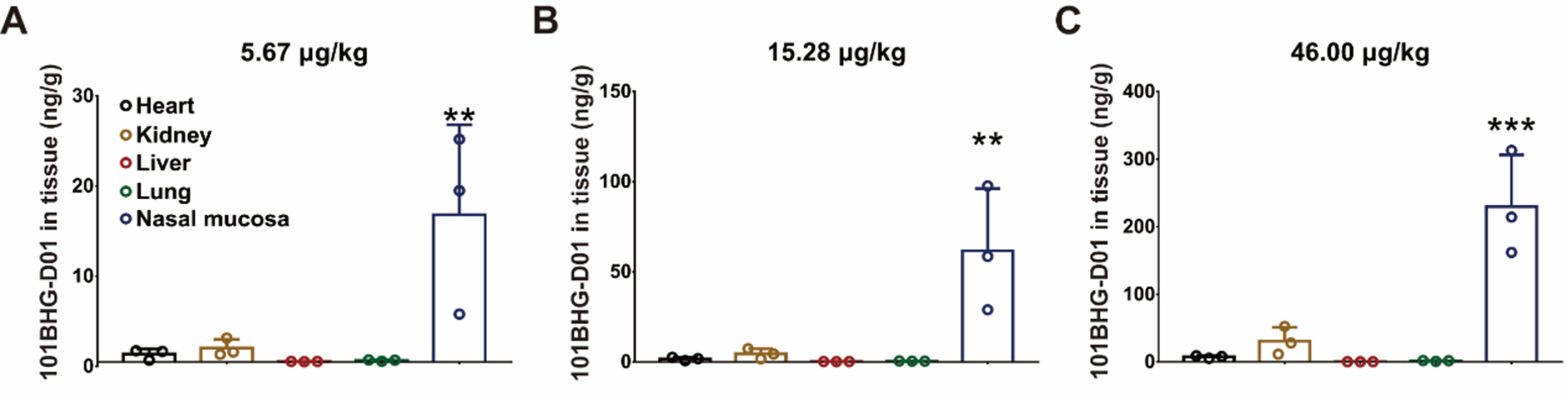
The residual drug concentration remains high in the nasal mucosa 24 h following nasal spray in dogs. **A**-**C**) 101BHG-D01 concentration in the nasal mucosa remains high 24 h after nasal spray. The results are shown as mean ± SD, n = 3/group. **p < 0.01, ***p < 0.001, compared with the nasal mucosa group, one-way ANOVA followed by Dunnett's multiple comparison tests.

To investigate the distribution of 101BHG-D01 in dogs, tissue samples were collected 24 h after nasal spray. We discovered that the nasal mucosa contained the highest concentration of 101BHG-D01 (heart, kidney, liver, and lung *vs*. nasal mucosa, 5.67 μg/kg: 862.3 ± 576.3 pg/g, 1,521.1 ± 975.7 pg/g, 45.9 ± 17.1 pg/g and 162.3 ± 75.6 pg/g *vs*. 16,332.0 ± 9,976.3 pg/g, *p* < 0.01; 15.28 μg/kg: 1,657.6 ± 919.5 pg/g, 4,463.2 ± 2,794.4 pg/g, 184.9 ± 59.0 pg/g, and 453.7 ± 69.1 pg/g *vs*. 61,683.0 ± 34,444.1 pg/g, *p* < 0.01; 46.00 μg/kg: 7,412.8 ± 1,829.9 pg/g, 30,565.2 ± 20,670.0 pg/g, 220.5 ± 81.3 pg/g, and 1,479.0 ± 604.3 pg/g *vs*. 229,751.8 ± 76,700.5 pg/g, *p* < 0.001; Figs. **4A**-**C**). The kidney had the second highest drug concentration compared to any other organs (5.67 μg/kg: 1,521.1 ± 975.7 pg/g; 15.28 μg/kg: 4,463.2 ± 2,794.4 pg/g; 46.00 μg/kg: 30,565.2 ± 20,670.0 pg/g; Figs. **4A**-**C**). Twenty-four h after administration, the drug concentration of 101BHG-D01 in the tissues or organs from high to low was ranked as follows: nasal mucosa, kidney, heart, lung, and liver.

### 3.3 Tissue Distribution of 101BHG-D01 in Rats Following Intranasal Administration

**Fig. (5):**
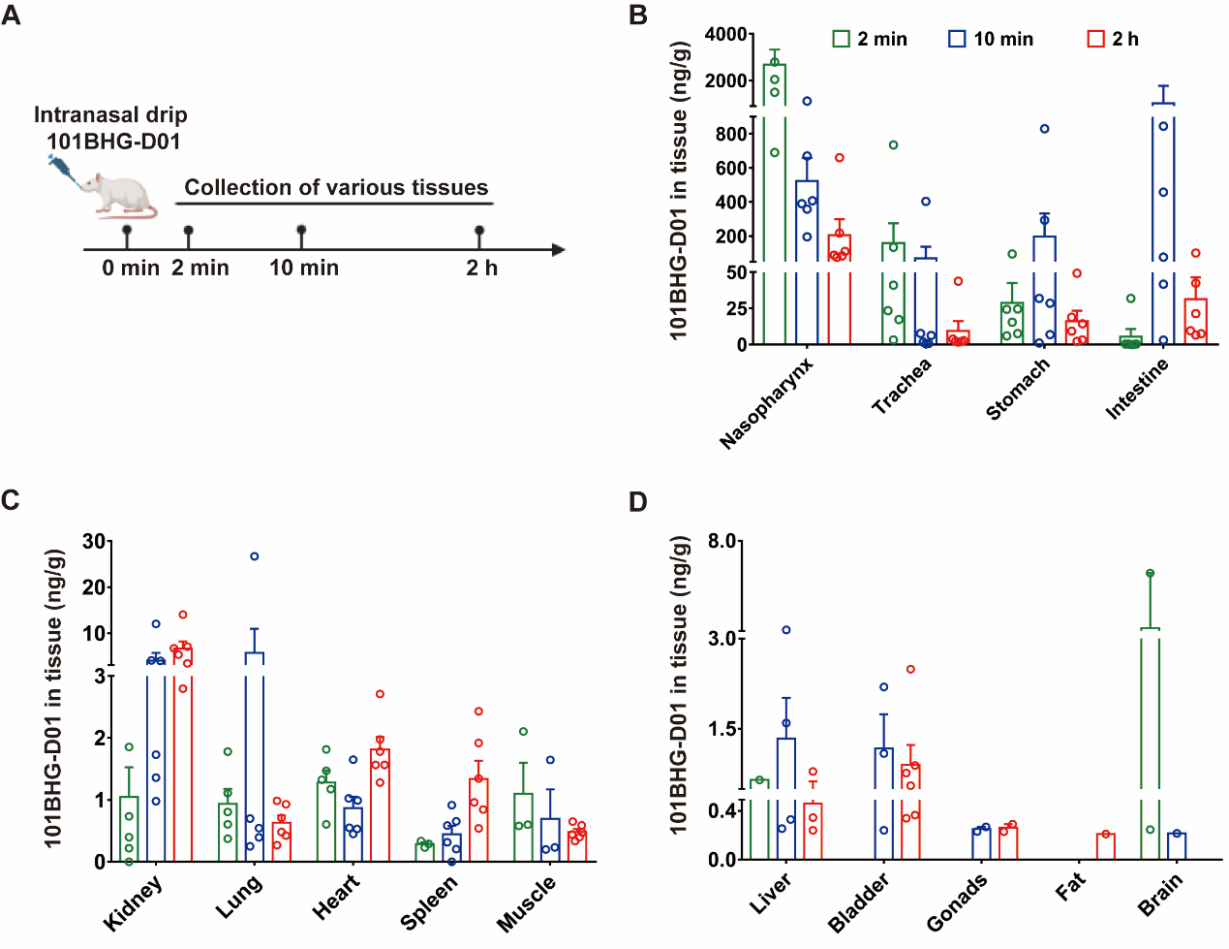
The organ distribution of 101BHG-D01 2 min, 10 min, and 2 h following intranasal administration in rats. **A**) Flow chart of the experiment. **B**-**D**) The quantification of 101BHG-D01 concentration indicates 101BHG-D01 concentrates in various organs 2 min, 10 min, and 2 h after intranasal administration. The results are shown as mean ± SD, n = 6/group.

To further investigate the distribution of 101BHG-D01 in rats, intranasal administration was used to simulate nasal spray administration (Fig. **5A**). 101BHG-D01 was mainly detected in the nasopharynx (2,678.33 ± 1,587.48 ng/g), trachea (159.09 ± 286.01 ng/g), stomach (28.86 ± 33.36 ng/g), intestine (8.28 ± 15.78 ng/g), remained low in the heart (1.28 ± 0.44 ng/g), kidney (1.25 ± 1.19 ng/g), muscle (1.10 ± 0.87 ng/g), lung (0.93 ± 0.54 ng/g), and was barely detectable in the liver, spleen, brain, fat, gonad (male: testis, female: ovary), and bladder 2 min following the administration. Ten min later, 101BHG-D01 concentration increased in the intestine (1,024.88 ± 1,841.46 ng/g), stomach (198.69 ± 328.65 ng/g), lung (5.72 ± 11.73 ng/g), kidney (4.05 ± 4.15 ng/g), liver (1.33 ± 1.36 ng/g) and spleen (0.54 ± 0.28 ng/g), decreased in the nasopharynx (523.58 ± 329.65 ng/g), trachea (70.20 ± 163.55 ng/g), heart (0.87 ± 0.46 ng/g) and muscle (0.69 ± 0.83 ng/g), and was almost undetectable in the bladder, brain, fat, and gonad. Two hours later, 101BHG-D01 concentration increased in the kidney (6.59 ± 4.04 ng/g), heart (1.81 ± 0.50 ng/g), and spleen (1.34 ± 0.71 ng/g), decreased in the nasopharynx (206.00 ± 228.57 ng/g), intestine (31.44 ± 36.68 ng/g), stomach (16.23 ± 17.40 ng/g), trachea (9.43 ± 16.88 ng/g), lung (0.63 ± 0.29 ng/g), muscle (0.48 ± 0.12 ng/g), and was undetectable in the liver bladder, brain, fat, and gonad. In conclusion, after intranasal administration, 101BHG-D01 was initially distributed in the nasopharynx and trachea, then accumulated in the intestine and stomach before excretion in rats (Figs. **5B**-**D**). Noticeably, 101BHG-D01 remained at a relatively high concentration in the nasopharynx even 2 h following the administration.

### 3.4 Characteristics of 101BHG-D01’s Primary Excretion in Rats

**Fig. (6):**
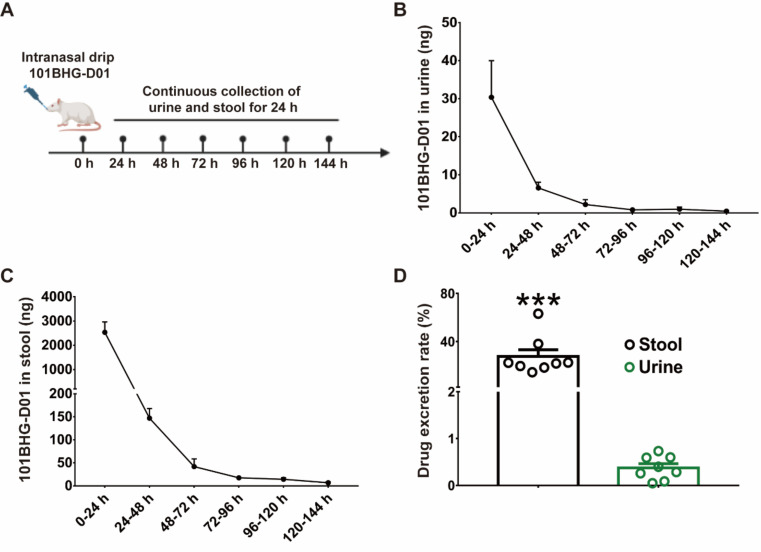
101BHG-D01 rapidly excretes in the urine and stool within 0-24 h in rats. **A**) Flow chart of the experiment. **B**) The accumulation of 101BHG-D01 in the urine after intranasal administration. **C**) The accumulation of 101BHG-D01 in the stool after intranasal administration. **D**) The cumulative excretion rate of 101BHG-D01 in the stool is higher than that in the urine after intranasal administration. The results are shown as mean ± SD, n = 8/group. ***p < 0.001, unpaired t-test with Welch's correction.

The primary mode of excretion of 101BHG-D01 in rats was investigated by collecting urine and stool at various time points following the intranasal administration (Fig. **6A**). The amount of drug excreted in the stool (intestinal excretion) and urine (renal excretion) gradually decreased over time. Between 0 h and 24 h, drug excretion peaked (urine: 30.37 ± 27.24 ng; stool: 2,536.26 ± 1,206.59 ng; Figs. **6B** and **C**). Drug excretion reached a nadir between 120 h and 140 h after our last detection (urine: 0.44 ± 0.48 ng; stool: 6.82 ± 5.40 ng; Figs. **6B** and **C**). By comparing cumulative excretion rates, we found that 101BHG-D01 excretion was at a significantly higher rate through the stool than in the urine (urine *vs*. stool, 0.38 ± 0.25% *vs*. 27.53 ± 15.97%, *p* < 0.001; Fig. **6D**).

## 4 DISCUSSION

Pharmacokinetic studies of novel drugs are critical to ensuring their safety and efficacy. To investigate the dynamic law governing the quantitative properties of 101BHG-D01's absorption, distribution, metabolism, and excretion *in vivo*, we investigated the pharmacokinetics of 101BHG-D01 in dogs and rats administered through the nasal spray and intranasal route, respectively. 101BHG-D01 was found to rapidly enter the bloodstream following nasal spray and have a long residence period in the body, with a plasma half-life at ~6 h. Additionally, we discovered that the pharmacokinetics of 101BHG-D01 was a linear kinetic process in dogs. By evaluating drug residues in tissues at various time points, we discovered that 101BHG-D01 retained a significant amount in the nasal cavity following nasal spray or nasal delivery. Finally, we discovered that 101BHG-D01 was primarily eliminated in the form of stool in rats.

We first investigated the dynamic regularity of 101BHG-D01's quantitative properties in dogs administrated by nasal spray. Consistent with previous reports, the drug rapidly reached the bloodstream following nasal administration [14]. Additionally, we observed that the drug's half-life in canine plasma was around 6 h. The term “plasma half-life” refers to the time necessary for a drug's plasma concentration to decrease by half, and its duration can indicate the rate of drug removal. It is a critical metric for clinically assessing the duration of the dosage interval and estimating the time required for the drug to be eliminated from the body during withdrawal. Moreover, we included additional critical pharmacokinetic characteristics, such as AUC, MRT, and CLz/F. These are critical references for developing and optimizing clinical trial dosage regimens.

Allergic rhinitis is a condition of the nasal mucosa caused by a multitude of circumstances, with the primary symptoms being sneezing and nose itching [15, 16]. At the moment, the nasal spray is the primary form of administration for allergic rhinitis treatment [14]. This is because it acts directly on the nasal mucosa tissue, achieving the goal of quick treatment. We discovered that 101BHG-D01 retains a high drug concentration in the nasal cavity for 2 min, 10 min, 2 h, and 24 h following nasal administration by comparing tissue samples taken at different time intervals and from different animals. However, the residual drug concentration may be ineffective at controlling disease symptoms beyond 24 h. Additionally, we discovered that 10 min after intranasal delivery, the gastrointestinal system had a greater drug concentration, which could be attributable to the increased rate of drug excretion during this time period. At the same time, this aspect was not disregarded that the drug may have adversely passed from the animal's oesophagus due to a shortcoming with the administration method. Additionally, we discovered that the concentration of the drug in the kidneys of animals rose over time, which could be due to drug excretion *via* the kidneys.

Due to the fast blood entry and tissue distribution characteristics, our nasal spray has a certain degree of permeability while simultaneously enhancing the retention of drugs in the nasal mucosa. Metoclopramide nasal spray [17], similar to our nasal spray, has been shown to have good permeability. We predict that 101BHG-D01 has a similar property; however, this needs to be studied further.

The process by which drugs are taken into the body and metabolites are expelled from the body is referred to as drug excretion. The process is inextricably linked to therapeutic efficacy, pharmacological efficacy duration, and toxic and adverse consequences. The goal of the research is to strike a balance between the efficacy and toxicity of drugs. The major routes of drug elimination in the body are renal excretion, intestinal excretion, bile excretion, and other excretion routes, such as saliva, milk, perspiration, and tears. We evaluated the two major modes of excretion in our trials (renal excretion and intestinal excretion). It was found that the fastest time period for drug excretion was between 0-24 h, which was also consistent with what was observed in tissue distribution. Finally, we discovered that 101BHG-D01 was primarily excreted in the intestinal system following intranasal administration in rats.

## CONCLUSION

The pharmacokinetics of 101BHG-D01 was found to be linear in dogs. 101BHG-D01 entered the bloodstream rapidly after nasal spray. Its plasma half-life was reported to be about 6 h. In addition, a large amount of 101BHG-D01 remained in the nasal cavity after nasal administration. Finally, 101BHG-D01 was cleared mainly in the form of feces in rats. In conclusion, we provide pertinent reference information regarding the design and optimization of drug delivery regimens for clinical trials.

## Figures and Tables

**Table 1:** Summary table of dosage groups and dosing schedules.

**Group**	**Dosage(µg/kg)**	**Dosing Concentration (mg/mL)**	**Route of Administration**
Low-dose group	5.67	0.14	Nasal spray
Medium-dose group	15.28	0.57	Nasal spray
High-dose group	46.00	1.71	Nasal spray

**Table 2:** Standard curves, correlation coefficients, linear ranges, and LLOQ of 101BHG-D01 in plasma of dogs and tissue samples of rats (n = 4).

**Samples**	**Equations of the Standard Curve**	**R**	**Liner Range (pg/mL)**	**LLQQ (pg/mL)**
Plasma^1^	y = 0.00189 x + 0.00824	0.9976	10-2,560	10
Plasma^2^	y = 0.0019 x + 0.00633	0.9996	10-2,560	10
Plasma^3^	y = 0.00198 x + 0.007	1.0000	10-2,560	10
Plasma^4^	y = 0.0018 x + 0.0162	0.9997	10-2,560	10
Tissue^1^	y = 0.000562 x + 0.000949	0.9999	40-10,000	40
Tissue^2^	y = 0.000585 x + 0.000791	0.9997	40-10,000	40
Tissue^3^	y = 0.00057 x + 0.0000879	0.9997	40-10,000	40
Tissue^4^	y = 0.00056 x + 0.000494	0.9996	40-10,000	40

**Abbreviations:** R: Correlation Coefficients; LLOQ: low Limit of Quantification.

**Table 3:** Precision and accuracy of HPLC–MS/MS method.

**Samples**	**Concentration (pg/mL)**	**Intra-day (n = 6)**		**Inter-day (n = 18)**	
		**RSD (%)**	**RE (%)**	**RSD (%)**	**RE (%)**
Plasma	10	7.38	-3.70	8.65	-3.70
	30	7.55	-5.07	8.89	-5.10
	200	2.23	-1.30	2.81	-1.30
	2,000	1.99	-3.70	4.49	-3.70
Tissue	40	4.00	-4.30	5.50	-4.30
	120	1.93	-2.77	2.70	-2.70
	960	3.43	-3.23	3.70	-3.20
	8,000	2.33	-1.87	2.40	-1.30

**Table 4:** Recovery of 101BHG-D01 in plasma of dogs and tissue homogenates of rats (n = 6).

**Samples**	**Concentration (pg/mL)**	**Extraction Recovery (%)**	**RSD (%)**
Plasma	30	95.50	4.49
	2,000	98.30	1.65
Tissue	120	95.05	3.20
	960	92.15	4.80
	8,000	94.74	2.50

**Table 5:** Stability of 101BHG-D01 in plasma of dogs, Tissue homogenates, and excrement of rats under different conditions (n = 3).

**Samples**	**Concentration (pg/mL)**	**Room Temperature RSD (%)**	**Freeze-Thaw Cycles RSD (%)**	**Long-Term Freezing at -70°C RSD (%)**
Plasma	30	9.54	1.35	8.99
	2,000	2.45	1.89	1.88
Tissue	120	2.60	5.10	2.00
	8,000	5.30	2.40	2.80
Urine	120	2.10	3.20	2.10
	8,000	1.50	1.10	1.50
Stool	120	8.90	4.70	5.40
	8,000	8.50	5.70	6.60

**Table 6:** Summary of pharmacokinetic parameters of 101BHG-D01 nasal spray in dogs.

**Pharmacokinetic Parameters**	**Unit**	**Low Group**	**Medium Group**	**High Group**
AUC (0-t)	ng/L∙min	22,412.37 ± 11,976.52	56,692.92 ± 28,333.99	291,752.70 ± 119,637.92
AUC (0-∞)	ng/L∙min	43,934.43 ± 28,291.80	62,469.57 ± 28,487.68	298,900.31 ± 121,971.43
R_AUC (t/∞)	%	67.70 ± 38.60	89.08 ± 10.37	97.60 ± 0.80
AUMC (0-t)	min∙min∙ng/L	2,466,189 ± 1,352,676	7,200,413 ± 4,204,734	44,315,366 ± 19,176,696
MRT (0-t)	min	107.97 ± 62.11	124.49 ± 15.84	147.04 ± 27.47
MRT (0-∞)	min	394.70 ± 383.90	230.13 ± 158.83	186.29 ± 46.17
VRT (0-t)	min^2	9,155.99 ± 12,105.36	13,349.79 ± 5,947.32	40,335.21 ± 21,581.07
VRT (0-∞)	min^2	325,478 ± 485,141	164,570 ± 301,632	120,982 ± 94,437
λz	1/min	0.006 ± 0.005	0.005 ± 0.004	0.003 ± 0.002
C_last	ng/L	39.62 ± 49.54	15.35 ± 4.74	16.66 ± 12.48
T_1/2z_	min	291.06 ± 309.71	257.65 ± 245.07	359.91 ± 208.75
Tmax	min	42 ± 13	33 ± 6	29 ± 11
Vz/F	L/kg	67.12 ± 91.61	111.51 ± 97.31	89.93 ± 45.03
CLz/F	L/min/kg	0.17 ± 0.06	0.34 ± 0.25	0.22 ± 0.19
Cmax	ng/L	166.75 ± 69.66	389.98 ± 192.68	2,371.91 ± 964.26

**Note:** According to the concentration-time curve, DAS3.0 software was used to calculate the pharmacokinetic parameters. Pharmacokinetic parameters of the low, medium, and high dose groups are displayed. The results are shown as mean ± SD, n = 6/group.

## Data Availability

The data that support the findings of this study are available from the corresponding authors upon reasonable request.

## LIST OF ABBREVIATIONS

AR: Allergic Rhinitis
IB: Ipratropium Bromide
SD: Sprague Dawley
M: Muscarinic
HPLC-MS/MS: High-Performance Liquid Chromatography-Tandem Mass Spectrometry
IS: Internal Standard
QC: Quality Control
RSD: Sample Relative Standard Deviation
RE: Relative Error
AUC: Area Under The Curve
MRT: Mean Residence Time
CLz/F: Clearance Rate
T1/2z: Half-Life

## References

[1] Waser P.G. (2011). The cholinergic receptor.. J. Pharm. Pharmacol..

[2] Nishtala P.S., Salahudeen M.S., Hilmer S.N. (2016). Anticholinergics: theoretical and clinical overview.. Expert Opin. Drug Saf..

[3] Panos R. (2013). Efficacy and safety of eco-friendly inhalers: focus on combination ipratropium bromide and albuterol in chronic obstructive pulmonary disease.. Int. J. Chron. Obstruct. Pulmon. Dis..

[4] Barnes P.J. (1993). Muscarinic receptor subtypes: implications for therapy.. Agents Actions Suppl..

[5] Lebois E.P., Thorn C., Edgerton J.R., Popiolek M., Xi S. (2018). Muscarinic receptor subtype distribution in the central nervous system and relevance to aging and Alzheimer’s disease.. Neuropharmacology.

[6] Calzetta L., Coppola A., Ritondo B.L., Matino M., Chetta A., Rogliani P. (2021). The impact of muscarinic receptor antagonists on airway inflammation: A systematic review.. Int. J. Chron. Obstruct. Pulmon. Dis..

[7] Tanahashi Y., Komori S., Matsuyama H., Kitazawa T., Unno T. (2021). Functions of muscarinic receptor subtypes in gastrointestinal smooth muscle: A review of studies with receptor-knockout mice.. Int. J. Mol. Sci..

[8] Soukup O., Winder M., Killi U.K., Wsol V., Jun D., Kuca K., Tobin G. (2017). Acetylcholinesterase inhibitors and drugs acting on muscarinic receptors- potential crosstalk of cholinergic mechanisms during pharmacological treatment.. Curr. Neuropharmacol..

[9] Schledwitz A., Sundel M.H., Alizadeh M., Hu S., Xie G., Raufman J.P. (2021). Differential actions of muscarinic receptor subtypes in gastric, pancreatic, and colon cancer.. Int. J. Mol. Sci..

[10] Saternos H.C., Almarghalani D.A., Gibson H.M., Meqdad M.A., Antypas R.B., Lingireddy A., AbouAlaiwi W.A. (2018). Distribution and function of the muscarinic receptor subtypes in the cardiovascular system.. Physiol. Genomics.

[11] Roth M. (2015). Airway and lung remodelling in chronic pulmonary obstructive disease: a role for muscarinic receptor antagonists?. Drugs.

[12] Leusch A., Eichhorn B., Müller G., Rominger K.L. (2001). Pharmacokinetics and tissue distribution of the anticholinergics tiotropium and ipratropium in the rat and dog.. Biopharm. Drug Dispos..

[13] Wood C., Fireman P., Grossman J., Wecker M., MacGregor T. (1995). Product characteristics and pharmacokinetics of intranasal ipratropium bromide.. J. Allergy Clin. Immunol..

[14] Laffleur F., Bauer B. (2021). Progress in nasal drug delivery systems.. Int. J. Pharm..

[15] Bousquet J., Anto J.M., Bachert C., Baiardini I., Bosnic-Anticevich S., Walter Canonica G., Melén E., Palomares O., Scadding G.K., Togias A., Toppila-Salmi S. (2020). Allergic rhinitis.. Nat. Rev. Dis. Primers.

[16] Braido F., Arcadipane F., Marugo F., Hayashi M., Pawankar R. (2014). Allergic rhinitis.. Curr. Opin. Allergy Clin. Immunol..

[17] Li Y., Fan X., Li W., Yang P., Zhang H., Tang D., Yin X., Sun J., Zheng A. (2018). Metoclopramide nasal spray in vitro evaluation and in vivo pharmacokinetic studies in dogs.. Pharm. Dev. Technol..

